# Supplementary material for: Characterization of humoral and SARS-CoV-2 specific T cell responses in people living with HIV
Source: Res Sq. 2021 Mar 17:rs.3.rs-309746. Preprint. [Version 1] doi: 10.21203/rs.3.rs-309746/v1 (PMC7987102; doi:10.21203/rs.3.rs-309746/v1)
Supplement: Supplement — ary Fig.1: Antigen binding screen and associations between humoral responses and cohort parameters. a Antigen binding screen in pre-pandemic samples from n=16 HIV positive donors and b the whole cohort with convalescent COVID-19 disease. Dotted lines indicate negative, low positive and positive threshold for absorbance [450nm]. c Correlation between age and S1 IgG titer according to gender in HIV negative donors and HIV positive subjects. d Correlation between age and ID50 according to gender in HIV negative donors and HIV positive subjects and e between DPSO and ID50 in the two study groups. f S1 IgG titer and ID50 levels summary dot plots according to ethnicity in HIV positive and negative donors. The non-parametric Spearman test was used for correlation analysis. *p < 0.05 Supplementary Fig.2: Magnitude of T cell responses and associations with HIV parameters, age, gender and ethnicity. a Percentage of responders to each peptide pools b Magnitude of the INF-γ-ELISpot responses. IFN-γ SFU/106 PBMCs are shown for SARS-CoV-2 ORF3a, ORF6, ORF7, ORF8 and Env between HIV negative (green) and HIV positive (red). (n=30 per group). c Magnitude of the total SARS-CoV-2 responses analyzed in pre-pandemic samples, confirmed SARS-CoV-2 and suspected cases with clinical definition but found to be SARS-CoV-2 seronegative on screening. In suspected cases, orange dots depict HIV negative and brown dots HIV positive donors. Correlation between CD4:CD8 ratio in HIV infected individuals with their d Nucleocapsid and e Membrane responses, depicting disease severity per donor. Correlation of total SARS-CoV-2 responses with age in f HIV negative and g HIV positive, depicting disease severity per donor. h Correlation of total SARS-CoV-2 responses with DPSO in HIV negative and i HIV positive, depicting disease severity per donor. j Magnitude of the total SARS-CoV-2 responses by ethnicity and k gender between HIV negative and HIV positive. The non-parametric Spearman test was use [file ca3a6c0a37515749f4b44426.pdf]

Supplementary Table 1. Cohort Demographics and Clinical Characteristics

		**HIV+, Lab confirmed SARS-CoV-2** RT-PCR+ and/or Ab Pos	**HIV+ *Suspected SARS-CoV-2**	**HIV- Lab confirmed SARS-CoV-2** RT-PCR+ and/or Ab Pos	**HIV- *Suspected/ Household**	**HIV+ pre-pandemic**
**Group size**	n	24	23	31	4	16
	SARS-CoV-2 seropositive at date of sampling, n (%)	23 (95.8)	7 (30.43)	29 (93.5)	0 (0.0)	0 (0.0)
**COVID-19 severity****	Asymptomatic (score 1), n (%)	2 (8.3)	0 (0.0)	4 (12.9)	1 (25.0)	-
	Non-hospitalized (score 2), n (%)	14 (58.3)	22 (95.7)	23 (74.2)	3 (75.0)	-
	Hospitalized (score 4-5), n (%)	8 (33.3)	1 (4.3)	4 (12.9)	0 (0.0)	-
**COVID-19 symptoms**	Fever, n (%)	13 (54.2)	18 (78.3)	16 (51.6)	2 (50.0)	-
	Shortness of breath, n (%)	14 (58.3)	14 (60.9)	12 (38.7)	1 (25.0)	-
	Fatigue, n (%)	21 (87.5)	20 (87.0)	20 (64.5)	4 (100.0)	-
	Cough, n (%)	17 (70.8)	18 (78.3)	12 (38.7)	3 (75.0)	-
	Headache, n (%)	10 (41.7)	14 (60.9)	13 (41.9)	2 (50.0)	-
	Altered taste/smell, n (%)	13 (54.2)	10 (43.5)	22 (71.0)	2 (50.0)	-
	Duration of symptoms in days, median (range)	14 (2-48)	21 (3-84)	10 (3-30)	7 (5-7)	-
	Days post-symptom onset (DPSO), median (range)	148 (46-232)	181 (126-273)	144 (101-220)	200 (125-203)	-
**Risk factors**	Age, median (range)	51 (30-73)	53 (30-67)	42 (26-65)	35.5 (30-41)	48 (30-60)
	Sex, n female:male:other	4:19:1	2:21:0	16:15:0	2:2:0	2:14:0
	BMI, median (range)	27.3 (20.2-33.8)	24.6 (20.3-39.4)	25.7 (19.1-37.7)	21.9 (20.0-27.6)	
**Ethnicity**	White, n (%)	16 (66.7)	17 (73.9)	19 (61.3)	1 (25.0)	11 (68.8)
	BAME, n (%)	8 (33.3)	6 (26.1)	12 (38.7)	3 (75.0)	5 (31.3)
**Smoking**	Current, n (%)	3 (12.5)	4 (17.4)	5 (16.1)	0 (0.0)	2 (12.5)
	Ex-smoker, n (%)	1 (4.2)	1 (4.3)	2 (6.5)	0 (0.0)	1 (6.3)
	Non-smoker, n (%)	20 (83.3)	18 (78.3)	24 (77.4)	4 (100.0)	13 (81.3)
**HIV parameters**	HIV viral load	<50	<50	-	-	<50
	CD4, median (range)	571 (133-1110)	586 (310-1360)	-	-	590 (350-940)
	CD4:CD8, median (range)	0.84 (0.17-2.54)	0.97 (0.37-2.15)	-	-	0.82 (0.43-2.26)
**Pre-existing conditions**	None, n (%)	10 (41.7)	8 (34.8)	15 (48.4)	4 (100.0)	11 (68.8)
	Diabetes, n (%)	6 (25.0)	1 (4.3)	1 (3.2)	0 (0.0)	1 (6.3)
	Hypertension/CVD, n (%)	7 (29.2)	2 (8.7)	3 (9.7)	0 (0.0)	2 (12.5)
	Renal disease, n (%)	2 (8.3)	2 (8.7)	0 (0.0)	0 (0.0)	0 (0)
	Respiratory disease (asthma and COPD), n (%)	2 (8.3)	4 (17.4)	1 (3.2)	0 (0.0)	0 (0)
	Liver disease, n (%)	1 (4.2)	2 (8.7)	0 (0.0)	0 (0.0)	1 (6.3)
	Other	ITP, psoriasis, pituitary gland failure	Osteoporosis, peripheral neuropathy, hypothyroidism	RA, iron deficiency, psoriasis, gout, SLE, hypothyroidism		

*Confirmed, possible and probable cases specified by the case definition for COVID-19, as of 29 May 2020, European Centre for Disease Prevention and Control [https://www.ecdc.europa.eu/en/covid-19/surveillance/case-definition]

**Severity of COVID-19 was classified according to the WHO (World Health Organisation) clinical progression scale (Aitken et al ., 2020).

Supplementary Table 2

<u>Fluorochrome-Conjugated Antibodies:</u>

Antibodies	Supplier	Identifier	Clone
APC anti-human IgM Antibody	BioLegend	Cat # 314510	Clone # MHM-88
APC/Cyanine7 anti-human CD19 Antibody	BioLegend	Cat # 363010	Clone # SJ25C1
APC/Cy7 anti-human CD197 (CCR7)	BioLegend	Cat # 353212	Clone # G043H7
Brilliant Violet 605™ anti-human CD3 Antibody	BioLegend	Cat # 317322	Clone # OKT3
Brilliant Violet 650™ anti-human CD127 (IL-7Rα) Antibody	BioLegend	Cat # 351325	Clone # A019D5
Brilliant Violet 650™ anti-human CD3 Antibody	BioLegend	Cat # 317324	Clone # OKT3
Brilliant Violet 711™ anti-human CD27 Antibody	BioLegend	Cat # 302833	Clone # O323
Brilliant Violet 785™ anti-human CD38 Antibody	BioLegend	Cat # 303530	Clone # HIT2
Alexa Fluor® 700 anti-human CD45RA Antibody	BioLegend	Cat # 304120	Clone # HI100
PE/Cyanine7 anti-human CD45RA Antibody	BioLegend	Cat # 304126	Clone # HI100
Brilliant Violet 421™ anti-human CD279 (PD-1) Antibody	BioLegend	Cat # 329920	Clone # EH12.2H7
PE/Dazzle™ 594 anti-human CD4 Antibody	BioLegend	Cat # 300548	Clone # RPA-T4
PE anti-human IgD Antibody	BioLegend	Cat # 348204	Clone # IA6-2
APC anti-human IFN-γ Antibody	BioLegend	Cat # 506510	Clone # B27
Brilliant Violet 785™ anti-human CD8a Antibody	BioLegend	Cat # 301046	Clone # RPA-T8
Brilliant Violet 711™ anti-human CD8a Antibody	BioLegend	Cat # 301044	Clone # RPA-T8
Brilliant Violet 510™ anti-human CD4 Antibody	BioLegend	Cat # 300546	Clone # RPA-T4
Brilliant Violet 510™ anti-human CD14 Antibody	BioLegend	Cat # 301842	Clone # M5E2
Brilliant Violet 510™ anti-human CD19 Antibody	BioLegend	Cat # 302242	Clone # HIB19
Brilliant Violet 711™ anti-human CD279 (PD-1) Antibody	BioLegend	Cat # 329928	Clone # EH12.2H7
Brilliant Violet 711™ anti-human PD-1 Antibody	BioLegend	Cat # 300232	Clone # RPA-2.10
PE/Cyanine7 anti-human CD154 Antibody	BioLegend	Cat # 310832	Clone # 24-31
APC-R700 Mouse Anti-Human CD196 (CCR6)	BD Biosciences	Cat # 565173	Clone # 11A9

BB515 Rat Anti-Human CXCR5 (CD185)	BD Biosciences	Cat # 564624	Clone # RF8B2
BV605 Mouse Anti-Human CD56	BD Biosciences	Cat # 562780	Clone # NCAM16.2
PC-Cy7 Mouse Anti-Human CD25	BD Biosciences	Cat # 335824	Clone # 2A3
BB700 Mouse Anti-Human CD16	BD Biosciences	Cat # 746199	Clone # 3G8
BB700 Mouse Anti-Human CD4	BD Biosciences	Cat # 566393	Clone # SK3
PE-Cy™5 Mouse Anti-Human CD183	BD Biosciences	Cat # 551128	Clone # 1C6/CXCR3
PE-Cy™5 Mouse Anti-Human HLA-DR	BD Biosciences	Cat # 562007	Clone # G46-6
FITC Mouse Anti-Human TNF-α	BD Biosciences	Cat # 554512	Clone # MAb11
GolgiStop (with Monensin)	BD Biosciences	Cat # 554724	
PerCP-eFluor 710 Anti-Human IL-2	eBioscience	Cat # 46-7029-42	Clone # MQ1-17H12
PerCP-eFluor 710 Anti-Human CD3	eBioscience	Cat # 46-0037-42	Clone # OKT3
PE Anti-Human TIGIT	eBioscience	Cat # 12-9500-42	Clone # MBSA43

Key Chemicals, Peptides, and Commercial Assays

Reagents	Supplier	Identifier	
PepTivator SARS-CoV-2 Prot_N	Miltenyi Biotec	Cat # 130-126-698	
PepTivator SARS-CoV-2 Prot_M	Miltenyi Biotec	Cat # 130-126-702	
PepTivator CMV pp65, human	Miltenyi Biotec	Cat # 130-093-438	
ProMixTM HIV Peptide Pool	Proimmune	Cat # PX-HIV	
Human IFN-γ ELISpot Kit	Mabtech	Cat # P3420-2A	
Human Anti-Cytomegalovirus IgG ELISA Kit (CMV)	Abcam	Cat # ab108724	
Brefeldin A	eBioscience	Cat # 00-4506-51	
Foxp3/TF Staining Buffer Set	Invitrogen	Cat # 00-5523-00	
BD Cytofix/Cytoperm™ Fixation/Permeabilization Solution Kit	BD Biosciences	Cat # 554714	

Fig. S1

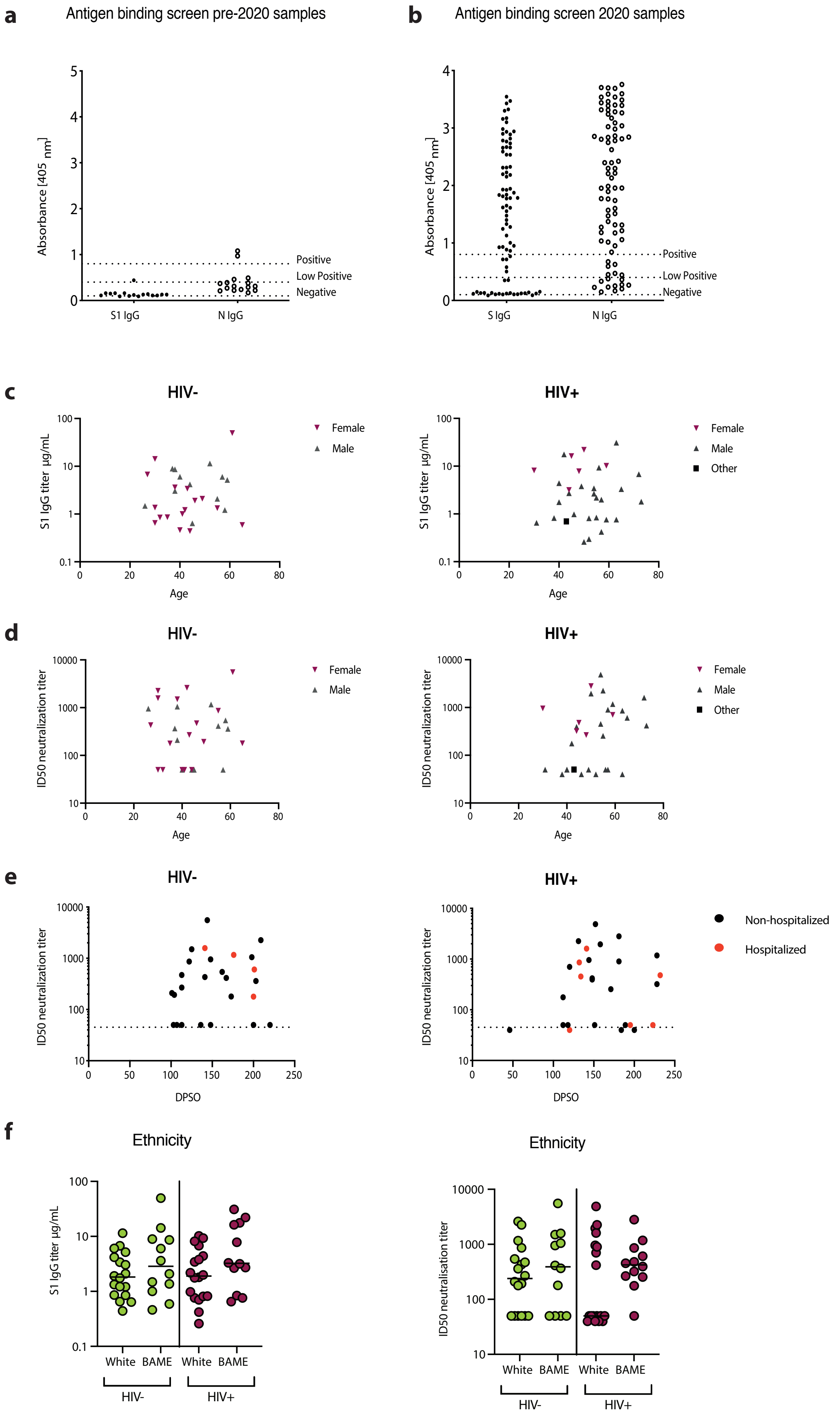
a
Antigen binding screen pre-2020 samples
Absorbance [405 nm]
Positive
Low Positive
Negative
S1 IgG
N IgG
b
Antigen binding screen 2020 samples
Absorbance [405 nm]
Positive
Low Positive
Negative
S IgG
N IgG
c
HIV-
S1 IgG titer µg/mL
Age
Female
Male
HIV+
S1 IgG titer µg/mL
Age
Female
Male
Other
d
HIV-
ID50 neutralization titer
Age
Female
Male
HIV+
ID50 neutralization titer
Age
Female
Male
Other
e
HIV-
ID50 neutralization titer
DPSO
HIV+
ID50 neutralization titer
DPSO
Non-hospitalized
Hospitalized
f
Ethnicity
S1 IgG titer µg/mL
White
BAME
White
BAME
HIV-
HIV+
Ethnicity
ID50 neutralisation titer
White
BAME
White
BAME
HIV-
HIV+

Fig. S2

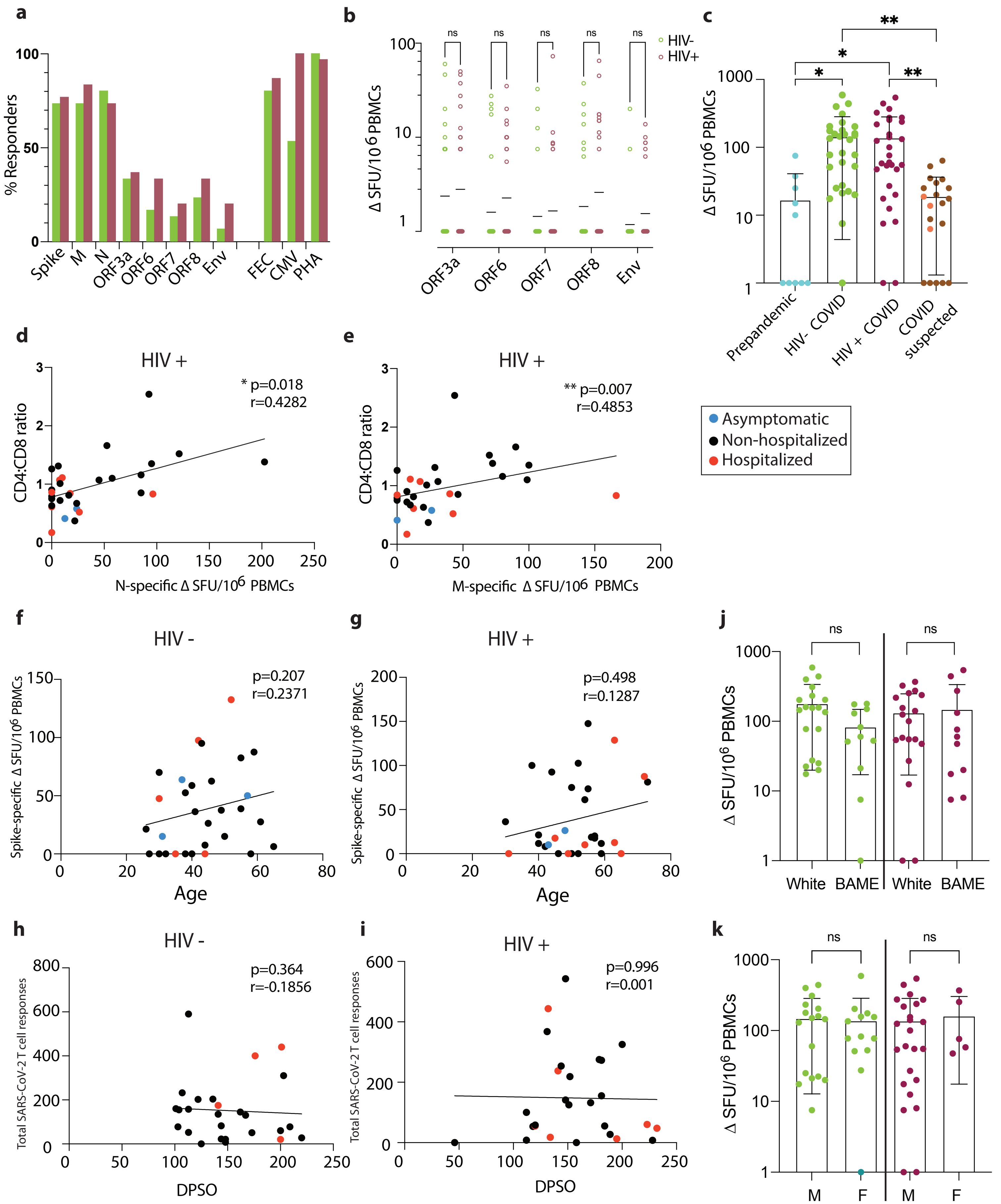

a
% Responders
Spike
M
N
ORF3a
ORF6
ORF7
ORF8
Env
FEC
CMV
PHA
b
Δ SFU/10^6 PBMCs
ns
HIV-
HIV+
ORF3a
ORF6
ORF7
ORF8
Env
c
Δ SFU/10^6 PBMCs
Prepandemic
HIV- COVID
HIV + COVID
COVID suspected
d
HIV +
* p=0.018
r=0.4282
CD4:CD8 ratio
N-specific Δ SFU/10^6 PBMCs
e
HIV +
** p=0.007
r=0.4853
CD4:CD8 ratio
M-specific Δ SFU/10^6 PBMCs
Asymptomatic
Non-hospitalized
Hospitalized
f
HIV -
p=0.207
r=0.2371
Spike-specific Δ SFU/10^6 PBMCs
Age
g
HIV +
p=0.498
r=0.1287
Spike-specific Δ SFU/10^6 PBMCs
Age
j
ns
Δ SFU/10^6 PBMCs
White
BAME
White
BAME
h
HIV -
p=0.364
r=-0.1856
Total SARS-CoV-2 T cell responses
DPSO
i
HIV +
p=0.996
r=0.001
Total SARS-CoV-2 T cell responses
DPSO
k
ns
Δ SFU/10^6 PBMCs
M
F
M
F

Fig. S3

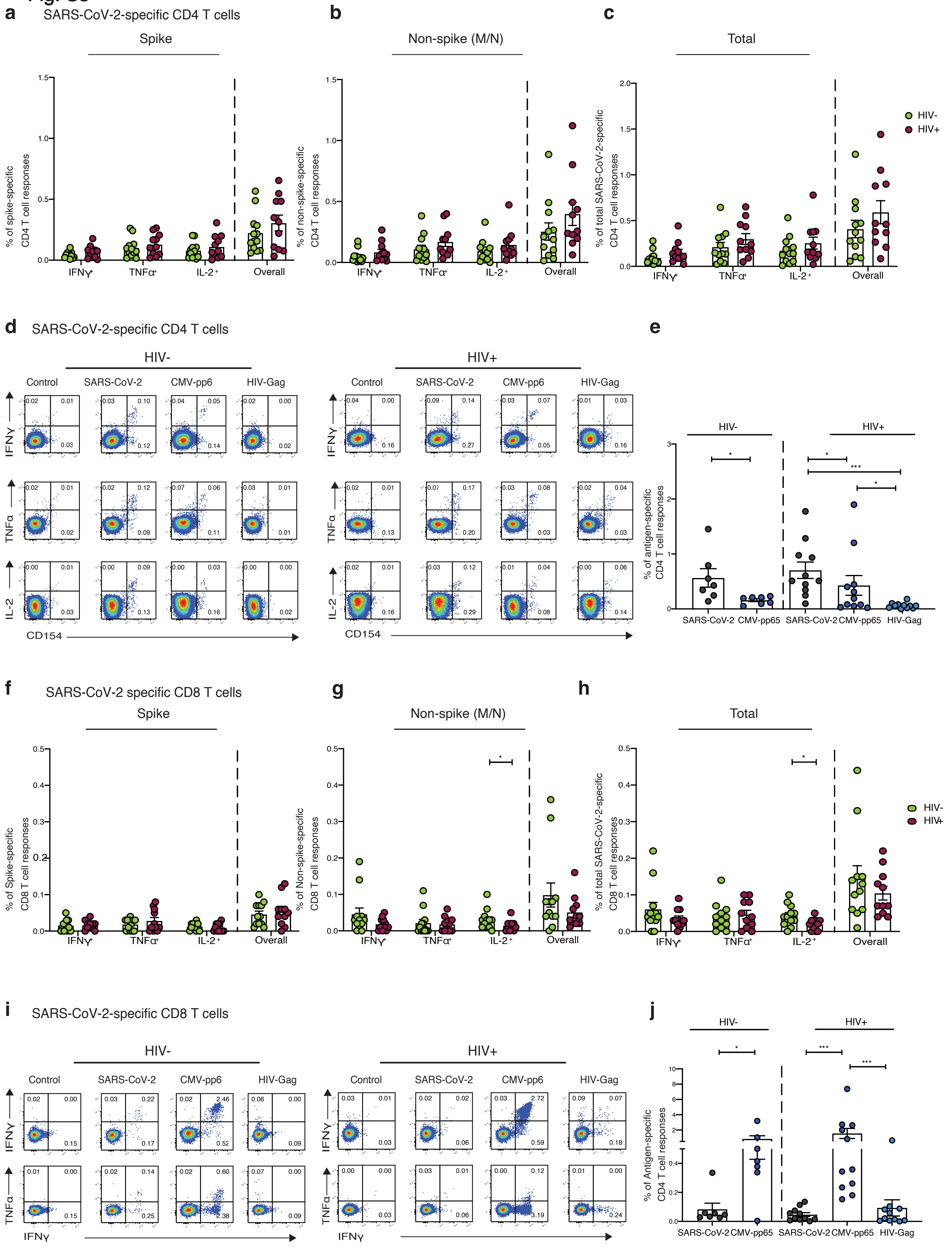
a
SARS-CoV-2-specific CD4 T cells
Spike
% of spike-specific CD4 T cell responses
IFNγ+
TNFα+
IL-2+
Overall
b
Non-spike (M/N)
% of non-spike-specific CD4 T cell responses
c
Total
% of total SARS-CoV-2-specific CD4 T cell responses
HIV-
HIV+
d
SARS-CoV-2-specific CD4 T cells
HIV-
HIV+
Control
SARS-CoV-2
CMV-pp6
HIV-Gag
IFNγ
TNFα
IL-2
CD154
e
% of antigen-specific CD4 T cell responses
SARS-CoV-2
CMV-pp65
HIV-Gag
f
SARS-CoV-2 specific CD8 T cells
Spike
% of Spike-specific CD8 T cell responses
g
Non-spike (M/N)
% of Non-spike-specific CD8 T cell responses
h
Total
% of total SARS-CoV-2-specific CD8 T cell responses
i
SARS-CoV-2-specific CD8 T cells
j
% of Antigen-specific CD4 T cell responses

Fig. S4

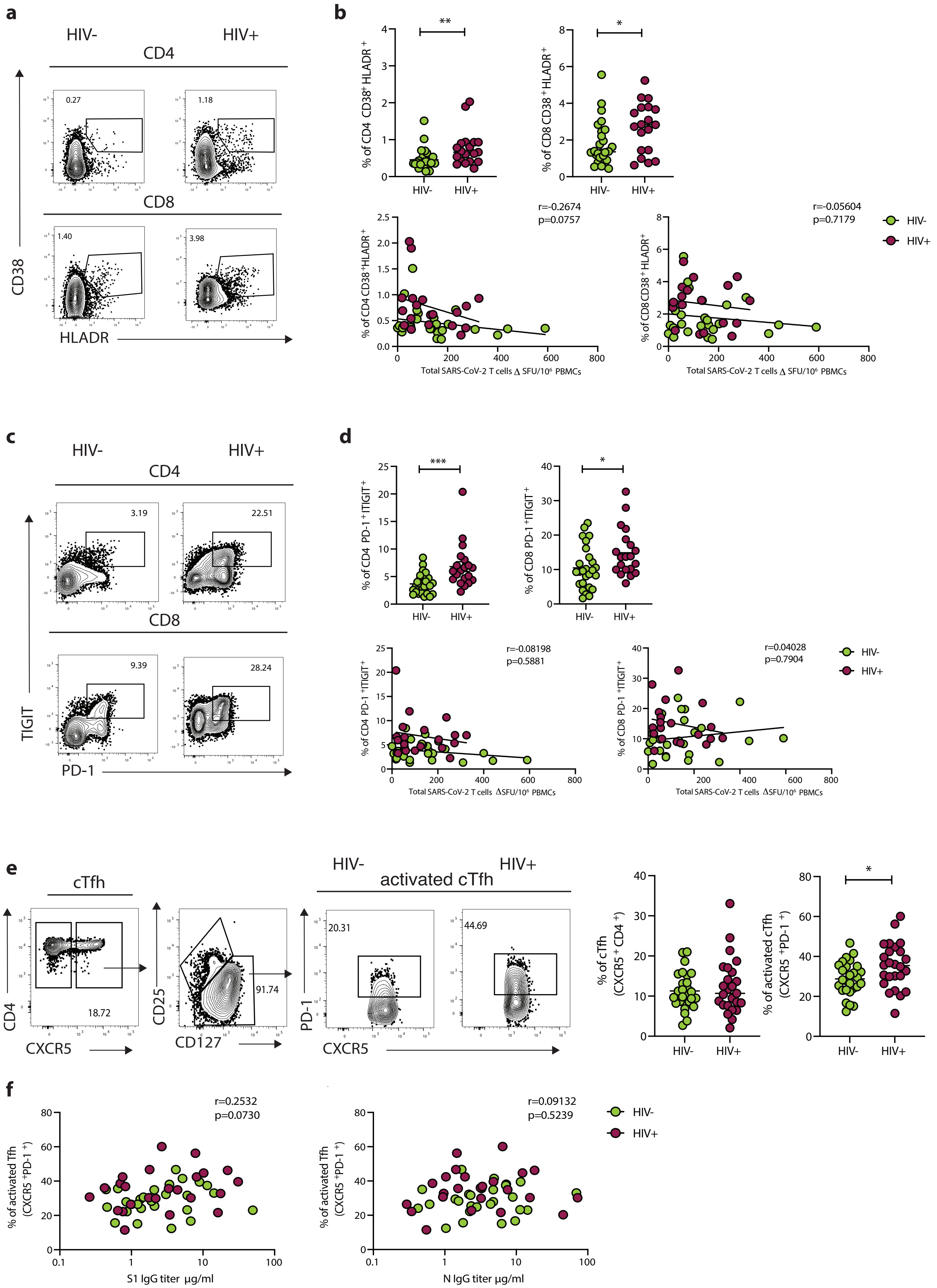

a
HIV-
HIV+
CD4
CD8
0.27
1.18
1.40
3.98
CD38
HLADR
b
% of CD4 CD38+ HLADR+
% of CD8 CD38+ HLADR+
**
*
HIV-
HIV+
r=-0.2674
p=0.0757
r=-0.05604
p=0.7179
Total SARS-CoV-2 T cells Δ SFU/10^6 PBMCs
c
HIV-
HIV+
CD4
CD8
3.19
22.51
9.39
28.24
TIGIT
PD-1
d
% of CD4 PD-1+ITIGIT+
% of CD8 PD-1+ITIGIT+
***
*
r=-0.08198
p=0.5881
r=0.04028
p=0.7904
Total SARS-CoV-2 T cells ΔSFU/10^6 PBMCs
e
cTfh
activated cTfh
HIV-
HIV+
18.72
91.74
20.31
44.69
CD4
CXCR5
CD25
CD127
PD-1
% of cTfh (CXCR5+ CD4+)
% of activated cTfh (CXCR5+PD-1+)
f
r=0.2532
p=0.0730
r=0.09132
p=0.5239
% of activated Tfh (CXCR5+PD-1+)
S1 IgG titer μg/ml
N IgG titer μg/ml
